# Prevalence of Human Papilloma virus in potentially malignant oral disorders is a risk factor for development of early dysplasia-A cytological investigation

**DOI:** 10.12669/pjms.40.1.7532

**Published:** 2024

**Authors:** Maham Javed, Rabia Anjum, Gulraiz Zulifqar, Farhan Rasheed, Ali Amar, Nadia Naseem

**Affiliations:** 1 Maham Javed, MBBS, MPhil Department of Morbid Anatomy & Histopathology, University of Health Sciences, Lahore, Pakistan; 2 Rabia Anjum, BDS, MPhil Department of Oral Pathology, University of Health Sciences, Lahore, Pakistan; 3 Gulraiz Zulifqar, BDS, FCPS Department of Maxillofacial, Jinnah Hospital Lahore, Pakistan; 4 Farhan Rasheed, MBBS, MPhil, FCPS Department of Microbiology, Allama Iqbal Medical College, Lahore, Pakistan; 5 Ali Amar, PhD Department of Human Genetics & Molecular Biology, University of Health Sciences, Lahore, Pakistan; 6 Nadia Naseem, MBBS, MPhil, PhD Department of Morbid Anatomy & Histopathology, University of Health Sciences, Lahore, Pakistan

**Keywords:** Potentially malignant oral disorders, Oral squamous cell carcinoma, Human papillomavirus, P16, Polymerase chain reaction, Early detection

## Abstract

**Objective:**

The proposed study was planned to screen Human Papilloma Virus (HPV) status in potentially malignant oral disorders (PMOD) and correlated HPV positivity with cytological changes in oral smears.

**Methods:**

This descriptive cross-sectional study was conducted at University of Health Sciences Lahore, Pakistan from April 2020 to April 2021. Oral smears from N=162 patients with PMODs were taken by the Cytobrush and Manual Liquid Based Cytology was performed followed by p16 antibody detection on immunohistochemistry and HPV-DNA detection by conventional polymerase chain reaction (PCR). The cytological changes were categorized according to the updated Bethesda Classification system 2014. SPSS was used to analyze data and p-Value of <0.05 was considered as statistically significant.

**Results:**

Out of total N = 162 patients, the most prevalent lesion [39% (n=63)] was lichen planus. Fifty six percent (n=90) of the patients were habitual chewers and 43% (n=70) were smokers. Pap staining of oral smears revealed atypical squamous cells of undetermined significance (ASCUS) in 45% (n=69) cases and in 2 % (n=4) of the samples diagnosis of atypical squamous cells-cannot exclude high-grade squamous intraepithelial lesion (ASC-H) was made. A total of 37% cases showed HPV positivity by polymerase chain reaction (PCR) while positive p16 expression was observed in 24% of the cases. ASC-H and ASCUS category showed significant association with HPV positivity (p=0.003).

**Conclusion:**

Early detection of PMODs by adopting minimally invasive cytological techniques and screening for HPV infection in local population is pivotal to reduce the morbidity and mortality associated with the advanced disease and carcinoma.

## INTRODUCTION

A wide variety of oral mucosal lesions with a more significant malignant potential is reported in the literature and are known as potentially malignant oral disorders (PMODs) comprising of oral leukoplakia, erythroplakia, erythroleukoplakia, oral submucous fibrosis (OSF), palatal lesions in reverse smokers, oral lichen planus, oral lichenoid reactions, graft vs. host disease, oral lupus erythematosus and few hereditary disorders such as dyskeratosis congenita and epidermolysis bullosa.1

Even though the drop in the prevalence of oral squamous cell carcinoma (OSCC) has taken place in the past three decades; this is most likely because of the knowledge of harmful effects of tobacco and alcohol usage still the incidence of OSCC in the oropharynx related to human papillomavirus (HPV) has risen primarily among younger men who have least or no tobacco and/or alcohol exposure.2 HPV-related oropharyngeal and oral malignancies display improved prognosis and are more responsive to treatment. Oral liquid-based cytology (LBC) has extensively substituted exfoliative cytology (EC) and is considered to be a highly acceptable and minimally invasive diagnostic tool for collecting characteristic cells from the oral mucosa.3

Moreover, by adopting this OLBC technique, added analyses can be carried out including immunocytochemical staining, molecular studies, DNA ploidy and assessment of mRNA expression.4 The p16 protein, also recognized as p16INKa, is a component of INK4 family of CDK inhibitors that functions in the cell cycle control. The subsequent amplified levels of p16 acts as prognostic indicator and surrogate marker for the transcriptionally active high-risk HPV and produces a target for immunohistochemistry.5

We, therefore, planned to categorize the oral cytological changes in PMODs according to updated Bethesda classification system 2014 and to explore the prevalence of HPV in these lesions. Also, the association between HPV and demographic as well as clinicopathological data, and characteristic risk factors comprising of tobacco and alcohol consumption in a Pakistani population was determined.

## METHODS

A descriptive cross-sectional study was performed from April 2020 to April 2021 comprising of total of N=162 patients with potentially malignant oral disorders reporting at Oral and Maxillofacial Department of Jinnah Hospital Lahore after taking written informed consent. Convenient sampling technique was used to collect the samples.

### Inclusion & Exclusion Criteria

In this study, patients of both genders with age group 18-60 years and clinically diagnosed cases of PMODs were included. However, patients with any diagnosed oral malignancy deranged clotting profile and inadequate specimens were excluded.

### Ethical Approval

This study was approved by Ethical Review Committee of University of Health Sciences Lahore Pakistan (UHS/REG-20/ERC/459). After taking written informed consent from the patients, the oral cavity was carefully examined by dental surgeons and characteristic appearances of the lesions were recorded in the proformas. Oral samples of each patient were taken by the Cytobrush (SurePath^®^). Three smears were prepared and the labeled slides were placed in fixative solution for 30 seconds.

After fixation, slides were air dried. The detachable head of Cytobrush was dropped in SurePath^®^ vial and the labeled SurePath^®^ vials were brought to the Department of Morbid Anatomy and Histopathology, University of Health Sciences Lahore for further processing. Smears were prepared through manual liquid based cytology (MLBC) which were then stained by rapid Papanicolaou stain and viewed under the microscope. The cytopathological findings were categorized according to the updated Bethesda classification system 2014 by three cytopathologists.6 Immunocytochemistry was performed by using p16 antibody (RMab) by Bio SB (Catalog No. BSB 3477) following the manufacture’s protocol and the antigen-antibody bonds were visualized using microscopy. Nuclear and cytoplasmic staining of infected cell containing HPV was noted and interpreted as positive. The percentage and intensity of p16-positive cells (cytoplasmic and nuclear) were analyzed through a mutual decision by three cytopathologists.

Total DNA was extracted from LBC samples using the QlAamp ® DNA Mini Kit from Qiagen, Cat #51304, according to the manufacturer’s protocol. The extracted DNA were assessed for quantity and quality (260/280 ratios) using NanoDrop 2000 spectrophotometer (Thermo Fisher Scientific, USA), whereas, β- actin housekeeping gene PCR amplifications were also performed to supplement the quality assessments of isolated DNA. PCR amplification of isolated genomic DNA was performed to get the desired amplified product of HPV.7 Briefly, L1 region of HPV genome (PCR product size of 150 bp) was amplified by conventional PCR using GP5+/GP6+ oligonucleotide primers: Forward 5’ TTTGTTACTGTGGTAGATACTAC 3’ and Reverse 5’ GAAAAATAAACTGTAAATCATATTC 3’. HPV primers were optimized on diagnosed cervical cancer samples. Positive and negative controls were cervical cancer and non-template samples, respectively. PCR products were electrophoresed on 2% agarose gels (BioWorld, USA) in TAE buffer along with 50bp and 100bp DNA ladder (Thermo Fisher Scientific, USA) and results were visualized on EZ Gel Documentation System (Bio-Rad, USA) which were recorded as positive or negative.

### Statistical analysis

The data was entered and interpreted using SPSS 25.0. Mean±SD given for quantitative variables. Frequencies and percentages were given for qualitative variables. Chi-square test was applied between qualitative variables. A p-Value of ≤0.05 was considered as statistically significant. Logistic regression was performed to predict the likelihood of the dependent outcome. Different associations among various variables were obtained including HPV status, oral Bethesda classification, patient demographic profile and chewing status. Criteria of p≤0.05 and 95% CI were set for all the tests.

## RESULTS

### Descriptive analysis

Out of total N = 162 patients the most prevalent lesion was lichen planus 39% (n=63) followed by leukoplakia 31% (n=51), oral submucous fibrosis is 24% (n=39) and erythroleukoplakia 6% (n=9). Most of the patients (n=63) were in age range 40-50 years while n=51 patients were in age range 51-60 years. Therefore, mean age of the patients was 44.98 ± 11.92 years. On analyzing gender, 65% (n=33) were females and 35% (n = 18) were males with OL, 43% (n=27) females and 57% (n=36) males with OLP, 54% (n=21) females and 46% (n=18) males with OSMF cases, 67% females (n=33) and 33% (n=18) males with ELP. When chewing habits of patients N=162 were evaluated, 56% (n=90) were habitual chewers while 44% (n=72) were non-chewers.

**Fig.1:**
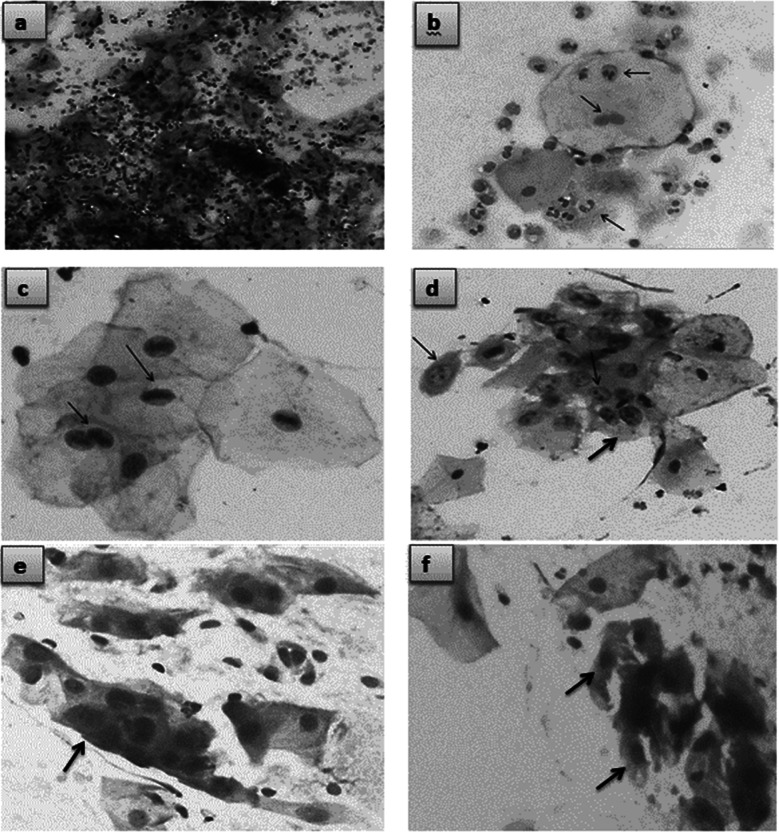
(a) Photomicrograph showing pap smear from a patient with NILM demonstrating acute inflammatory exudate with normal cellular features (20X; Pap stain) (b) Photomicrograph of pap smear showing NILM category with neutrophilic infiltration and binucleation (arrows) in superficial squamous cells (40X; pap stain) (c) Photomicrograph of pap smear with ASCUS category showing increased mitotic figures (arrows) in superficial squamous cells (40X; pap stain) (d) Photomicrograph of pap smear with ASCUS category showing moderate pleomorphism and prominence of nucleoli (arrows) in superficial squamous cells (40X; pap stain) (e & f) Photomicrograph of pap smear with ASC-H category showing marked hyperchromasia, increased N/C ratio and irregular nuclear contours (40X; Pap stain).

Among all the patients, 43% (n=70) were smokers and majority of them (n=46) used to smoke > 20 cigarettes/ day. Pap staining of oral smears revealed 55% (n=89) lesions as negative for intraepithelial lesion or malignancy (NILM) showing reactive cellular changes with mild to moderate inflammatory cells infiltration predominantly neutrophils was found in this category. Among rest of the 45% (n=69) cases, diagnosis of atypical squamous cells of undetermined significance (ASCUS) [43% (n=69)] and atypical squamous cells-cannot exclude high-grade squamous intraepithelial lesion (ASC-H) [2 % (n=4)] were made (Fig.1 a-f).

**Fig.2:**
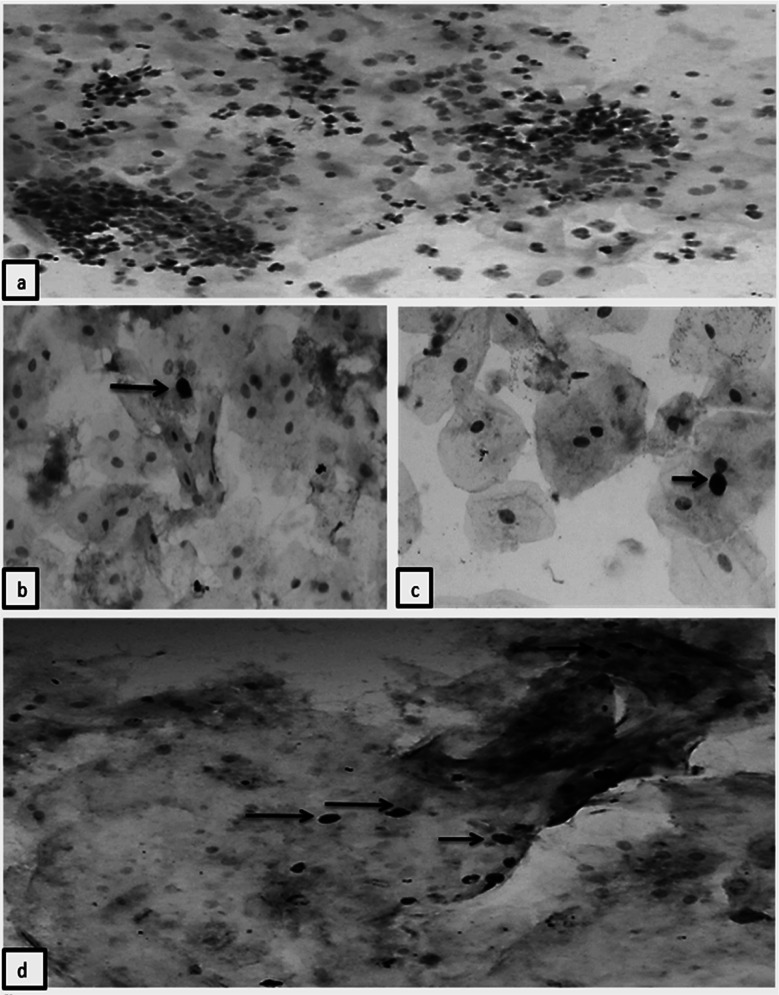
(a) Photomicrograph of ICC showing nuclear staining of sheets and clusters of neutrophils (arrows) by p16 antibody (20X) (b) Photomicrograph of ICC showing nuclear and peri-nuclear staining of squamous cell (arrow) by p16 antibody (40X) (c) Photomicrograph of ICC showing nuclear and cytoplasmic staining of neutrophil (arrows) by p16 antibody (40X) (d) Photomicrograph of ICC showing nuclear p16 staining of superficial squamous cells (arrows) (20X).

**Fig.3:**
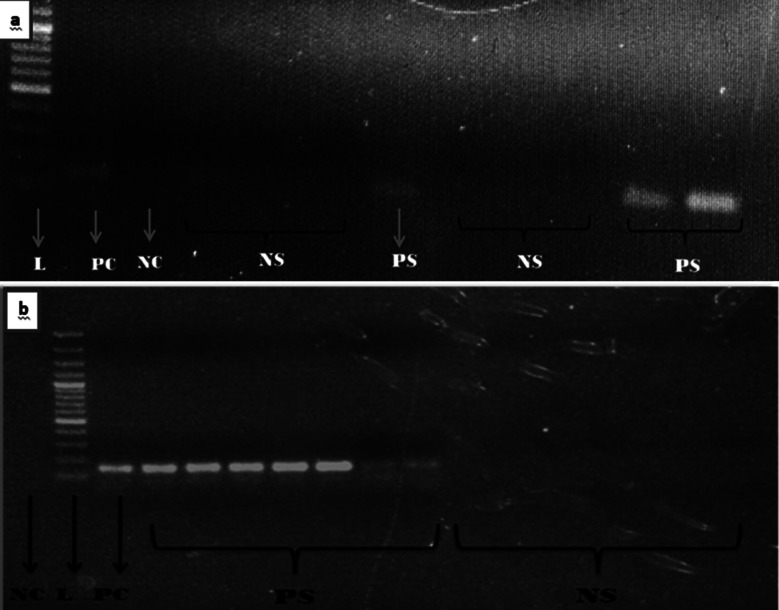
(a-b): Agarose gel electrophoresis for PCR amplification of HPV GP5+/6+ showing the presence of HPV-DNA (150bp amplicon) in samples. NC=negative control, PC=positive control (cervical cancer), L=100bp ladder, PS=positive samples, NS=negative samples.

The demographic profile, socio-economic status (SES) and chewing habits in N = 162 patients were descriptively analyzed (Table-I). HPV p16 antibody staining was positive in the nuclei of superficial and intermediate squamous cells. In addition, the nuclei of neutrophils also stained positively for the antibody thus demonstrating viable antigenic reservoir (Fig.2 a-d). Among n=4 ASC-H cases, n=3 (75%) cases came out to be HPV positive. These patients had history of dual smoking for > five years and were in 36-45 years old category. The current study showed 37% (n=60) HPV-DNA positive samples by conventional PCR using GP5+/GP6+ primers (Fig.3 a-b).

**Table-I:** Descriptive analysis for demographics, socioeconomic status (SES) and clinicopathological features of PMODs, N=162

Sr.No.	Demographic Characteristics	Frequency (n)	Percentage (%)
	***Gender***		
	Female	87	54
	Male	75	46
	Mean age of the patients = 44.98 ± 11.92 years		
	***Occupation status***		
	Employed	91	56
	Unemployed	71	44
	***Type of occupation***		
	Labourer	71	44
	Driver	35	22
	Guard	32	19
	Tailor	24	15
	***Monthly income of the family***		
	Less than $120(<PKR 20,000)	144	89
	$120-250 (PKR 20,000-40,000)	18	11
	$250-380 (PKR 40,000 – 60,000)	0	0
	More than $380 (>PKR 60,000)	0	0
6.	***Smoking History***		
	Smokers	70	43
	Non-smokers	92	57
7.	***Frequency of cigarette smoking per day***		
	< 5 cigarettes per day	3	2
	5-20 cigarettes per day	21	13
	>20 cigarettes per day	46	28
8.	***Chewing Addiction***		
	Yes	91	56
	No	71	44
9.	***Type of smokeless tobacco chewing***		
	Betel quid (paan)	81	50
	Areca Nut (chalia)	36	22
	Naswar	15	9
10.	***Clinicopathological features***		
i.	***Reduction in mouth opening***		
	Yes	49	30
	No	113	70
ii.	***Burning sensation in mouth***		
	Yes	150	93
	No	12	7
iii.	***Discoloration of Buccal Mucosa***		
	Yes	110	68
	No	52	32
11.	***Site of lesion***		
	Buccal mucosa	110	68
	Non-buccal mucosa	52	32
12.	***Lesion type***		
	Leukoplakia	51	32
	Lichen Planus	64	39
	Oral submucous fibrosis	38	24
	Erythroleukoplakia	9	5
13.	***HPV status***		
	p16 positivity by ICC	39	24
	P16 positivity by PCR	60	37
14.	***Bethesda Classification***		
	NILM	89	55
	ASCUS	69	43
	ASC-H	4	2

***Abbreviations:*** ASCUS- Atypical squamous cells of undetermined significance, ASC-H- Atypical Squamous Cells, Cannot Rule Out High Grade Squamous Intra-epithelial Lesion, ICC- Immunocytochemistry, NILM- Negative for intraepithelial lesion or malignancy, PCR- Polymerase Chain Reaction.

### Association analysis

Chi-square test was performed to observe the association between HPV status and Bethesda category. Statistically significant relation was found between HPV positivity and Bethesda category with (*p*=0.002). Majority of the samples n=42 (60.9%) belonged to ASCUS and ASC-H category and were HPV-DNA positive. Out of n=4 ASC-H cases, n=3 (75%) cases were HPV-positive. The significant association between the demographic variables such as gender, age, education status, occupation (p <0.05, OR>1) as well as HPV status by PCR and ICC was checked with Bethesda Category (NILM, ASCUS and ASC-H). In the univariate regression analysis, presence of HPV both by PCR and ICC showed significant association with Bethesda category (p=0.003 and 0.03, respectively) (Table-II). Moreover, all of the n=4 ASC-H cases belonged to the age category of 36-45 years and were habitual dual smokers (tobacco and smokeless tobacco). No significant association was found between gender, age, education status and occupation with chewing habit.

**Table-II:** Univariate and multivariate logistic regression analysis of Bethesda Category, demographic profile and HPV status

Factors	Bethesda Category		OR (95% CI)	Adjusted OR (95% CI)

	NILM n (%)	ASCUS & ASC-H n (%)		
***Gender***				
Female	51 (58.6)	36 (41.4)	1^*^	
Male	42 (56)	33 (44)	0.89 (0.35-2.65) (*p*=0.898)	
***Age (in years)***				
18-25	24 (89.9)	3 (11.1)	1^*^	
26-35	3 (33.3)	6 (66.7)	0.161 (0.016-1.606) (*p*=0.120)	
36-45	18 (42.9)	24 (57.1)	2.571 (0.192-3.447) (*p*=0.476)	
46-50	21 (58.3)	15 (41.7)	1.714 (0.403-7.292) (*p*=0.466)	
51-60	27 (56.2)	21 (43.8)	0.918 (0.202-4.175) (*p*=0.912)	
***Education Status***				
Un-Educated	48 (64)	27 (36)	1^*^	
Educated	45 (51.7)	42 (48.3)	0.603 (0.202-1.80) (*p*=0.603)	
***Occupation***				
Un-Employed	42 (58.3)	30 (41.7)	1 ^*^	
Employed	51 (56.7)	39 (43.3)	0.934 (0.315-2.76) (*p*=0.092)	
***HPV-PCR***				
Negative	75 (73.5)	27 (26.5)	1^*^	
Positive	18 (30)	42 (70)	0.154 (0.045-0.52) (*p*=0.003*)	0.133 (0.013-1.341)
***HPV- ICC***				
Negative	81 (65.9)	42 (34.1)	1^*^	
Positive	12 (30.8)	27 (69.2)	0.230 (0.06-0.883) (*p*=0.03*)	0.712(0.062-8.223)

1* is taken as reference. P ≤ 0.05 was considered significant. Abbreviations: NILM-Negative for intraepithelial lesion or malignancy, ASCUS- Atypical squamous cells of undetermined significance, ASC-H- Atypical Squamous Cells, Cannot Rule Out High Grade Squamous Intra-epithelial Lesion.

## DISCUSSION

Despite substantial advances in the cancer management, timely detection of oral carcinoma and its curable precursor lesions remains the soundest way to certify patient’s survival and better-quality of life.8 Tobacco and alcohol consumption are reflected as the key risk factors for OSCC occurrence, while human papilloma virus (HPV) infection is evolving as the foremost risk factor in the malignancies of oral cavity and oropharynx. The prevalence of oral HPV infection has markedly increased in the last decade, raising fears about HPV role in the malignant transformation of PMODs. However, the relationship between HPV infection and PMODs is still controversial.9 Out of the total N=162 patients in this study, 54% (n=87) were females and 46% (n=75) were males. A study on PMODs conducted by Queiroz et al.10 also showed female predominance (57%) as compared to males which may be due to increase in chewing habits in female population over the years.

The descriptive analysis of the present study showed increased prevalence of PMODs among females with a mean age of 44.8 ± 11.92 years. Maximum number of the patients (n=48) affected were between 51-60 years of age which is in favor with the studies by Pires et al.11 and Villa et al.12 who concluded that elevated risk of developing PMODs is related to an increasing age. Lichen planus was the most prevalent lesion in the present study comprising 39% of the total patients. Various studies have shown leukoplakia to be most prevalent followed by other PMODs.13-15 A study by Kotnis et al.16 concluded OSMF to be most frequent of all PMODs (32%) whereas lichen planus was found to be most common lesion in the study reported by Al-Maweri et al.17 This variation in the prevalence of different PMODs is likely because of the differences in their risk potential and etiological factors.

As per the present study, 54% of the patients had smoking propensity. Out of these 54% patients who smoke, 55% were double consumers (Smoking and SLT). These outcomes are in consistence with various studies on PMODs showing similar findings concluding that concomitant smoking and SLT consumption exceedingly increases the incidence of oral lesions and oral malignancy.17,18

Cytological diagnosis of N=162 cases were made based on the updated Bethesda system 2014. In the present study NILM was found to be commonest (55%) finding followed by ASCUS (43%) and ASC-H (2%). Majority (93%) of OSMF cases showed presence of micronuclei, 95% of the OLP cases exhibited hyperkeratosis whereas 100% cases of erythroleukoplakia showed an increased mitosis, binucleation, moderate nuclear pleomorphism and prominence of nucleoli. Significant association (p-Value ≤ 0.05) was present between leukoplakia and increased mitotic rate, OSMF and micronuclei formation and lichen planus and hyperkeratosis. Literature has shown similar microscopic findings having significant association between hyperkeratosis and lichen planus; increased mitotic rate and pleomorphism with leukoplakia as well as erythroleukoplakia and micronuclei formation and hyperkeratosis with OSMF.19,20

In the present study, logistic analysis reveals significant association (p=0.003 and 0.03, respectively) between Bethesda category (dependent variable) and HPV positivity. Moreover, present study shows 3/4 ASC-H and 56% (n=39) ASCUS cases were HPV positive. When univariate and multivariate logistic analysis was applied between HPV status (dependent variable) and demographic profile, no significant association (p > 0.05) was found. Similar results were obtained by a recent study in which lichen planus showed the highest HPV infection rates (75%) followed by OL cases (33.3%).21 A study by Ramaya et al,22 showed 20% of the OL cases to be HPV positive by PCR and all were habitual smoke/smokeless tobacco chewers. However, no significant association was found between HPV positivity and increased risk of OL.

In the current study, 37% (60/162) cases were HPV positive detected by conventional PCR while 24% (39/162) cases came out to be HPV positive by ICC. Literature has also shown high p16 sensitivity (94%) and low specificity (82%) as compared to PCR/ISH mainly due to subjective interpretation associated with weak/equivocal staining resulting in high amounts of false-positive cases which makes ICC technique unreliable in these situations.23,24 Pathak et al.25 determined HPV status of the tumors using HPV-DNA PCR and p16 immunostaining in oral dysplasia and oral carcinoma in 50 patients, out of which 83% HPV DNA positive cases by conventional PCR were also positive for p16 expression. Practicing two-step approach including p16 IHC followed by HPV DNA detection by either PCR or ISH has been emphasized in the literature.

### Limitations

There were some cost-related issues as SurePath is an expensive technique therefore; repetitive sampling by adopting trouble-shooting methods couldn’t be done. Moreover, it was not possible to do the follow-up due to failure of the patients to cooperate throughout the study.

## CONCLUSION

The early detection of PMODs by adopting advanced minimally invasive techniques and screening for HPV is of vital importance as the chances of survival remarkably increase when these lesions are detected at their early stages of development.

### Recommendations

PMODs are often detected in their advanced stages and their delayed diagnosis halts the successful management of these lesions resulting in their malignant transformation. The screening for these PMODs using cytology-based techniques can assist as a valuable addition and a capable cost-effective tool in a poor-resource set-up. Moreover, for the detection of HPV, there is an essential need for a distinct modality test to enhance the extensive adoption and reduce the expenditures.

Hence, long-duration and extensive studies are essential to comprehend the characteristic outcome of PMODs which in return may facilitate the timely diagnosis of these lesions and render an adequate management, thereby reducing the morbidity and the mortality related to the malignant transformation. This can only be achieved through persistent and focused interdisciplinary-efforts.

### Authors’ Contributions:

**MJ:** Conception and drafting of manuscript; data acquisition, interpretation.

**RA:** Drafting of manuscript; data acquisition and interpretation Co-supervision of work.

**GZ:** Data acquisition and interpretation, revising the manuscript critically for important intellectual output (consultant oral maxillofacial surgeon).

**FR:** Microbial aspects of handling cytological samples (consultant microbiologist).

**AA:** Molecular component of the study.

**NN:** Conception of study, revising the manuscript critically for important intellectual output, complete supervision of all the pathological techniques, diagnosis and collaboration with clinical and molecular data. Final approval of the version of the manuscript to be published;

All authors are responsible and accountable for the accuracy and integrity of the work.
